# The Experiment Station Veterinarian as a Member of the State Board of Health1Read before the Thirty-fifth Annual Meeting of the United States Veterinary Medical Association, September, 1898.

**Published:** 1899-02

**Authors:** M. H. Reynolds

**Affiliations:** St. Anthony Park, Minn.


### THE EXPERIMENT STATION VETERINARIAN AS A MEMBER OF THE STATE BOARD OF HEALTH.[1]

By M. H. Reynolds, D.V.M.,
ST. ANTHONY PARK, MINN.

It is unfortunate that there is not greater uniformity in methods of controlling infectious diseases among domestic animals. Some States have adopted the plan of a State veterinarian, assisted by local deputies, the State veterinarian having little or no connection with the State Board of Health; while other States are trying to control infectious diseases among domestic animals through boards of livestock commissioners. Some States have a State veterinarian working on very meagre salary, and other States have State veterinarians who are non-graduates and who are given considerable authority.

And still other States are trying to control these diseases by means of official titles—*i.e.*, they have officers and titles, but these officers are practically without funds and without sufficient authority.

In Minnesota all police authority concerning infectious diseases of animals is vested in the State Board of Health. Until January 1, 1897, this board was composed exclusively of physicians. For a great many years Minnesota's State Board of Health presented the strange combination of a board composed exclusively of practitioners of human medicine, having absolute authority concerning infectious diseases of domestic animals. During that time the gentleman who held the position of experiment station veterinarian was expected to visit outbreaks and accomplish marvellous things in the way of checking infectious diseases, without any authority. This situation and the results of this method did not prove satisfactory

[1] Read before the Thirty-fifth Annual Meeting of the United States Veterinary Medical Association, September, 1898.

to our stock interests. Stockmen made such vigorous objections during the winter and spring of 1896 and 1897 that the Governor decided to appoint a veterinarian to membership on the State Board of Health. After due consideration he appointed the experiment-station veterinarian. This is the present situation in our State. It is quite possible that another veterinarian may be appointed to membership on the board in the future, and then the work will be divided more nearly as it should be.

Our newly appointed member of the State Board of Health was soon made chairman of the committee on infectious diseases of animals, and given immediate charge of the correspondence and general office work pertaining to infectious diseases of domestic animals. After about six months of this work he was made director of a newly created veterinary department. This divided the work of the board into three parts: that of the secretary and general executive officer, the bacteriological laboratory in charge of a director (and, by the way, we have a laboratory and bacteriologist in connection with this work in Minnesota, of which we are proud), and the third department is known as the veterinary department. Rules which partly define the duties and authority of the director of the veterinary department have been adopted as follows:

RULES CONCERNING WORK IN THE VETERINARY DEPARTMENT.

1. The director of the veterinary department shall have the privilege of proposing such circulars and rules as he may deem necessary for the purpose of defining the policy of the board with reference to the veterinary work of the board. Such circulars and rules shall be submitted to the executive committee or to the State Board of Health, for approval.

2. The director shall conduct the correspondence dealing exclusively with veterinary matters. He shall have the necessary police authority to enable him to order quarantine when in his judgment such course is necessary. He shall have authority to use his judgment in releasing quarantine in unusual cases independently of the rules governing quarantine.

3. All agents and employés doing veterinary work in the field shall report to the director, and it shall be the duty of the director to furnish the secretary with such summaries of regular work and with such other information as the secretary may need.

4. It shall be the duty of the director to refer such matters as violation of the law dealing with infectious diseases of animals, general enforcement of said law, and indifference and carelessness of local health-officers, to the secretary for action.

5. It shall be the duty of the field-veterinarian to investigate outbreaks of infectious diseases among domestic animals when deemed advisable by the director of the veterinary department, and to attend to such experimental and other veterinary work as may seem necessary. When not doing field-work, it shall be his duty to assist the director in correspondence and other office-work.

6. The field-veterinarian shall have authority to order quarantine, to kill and release quarantine of domestic animals, in accordance with the rules and recognized methods of the State Board of Health.

7. It is hereby declared the policy of the State Board to pay the salary and furnish transportation for the field-veterinarian. Local boards are expected to pay all his other legitimate expenses incurred in work for them.

The work of the veterinary department has grown rapidly in all directions. During the last year we employed one field-veterinarian. This spring we added another. Thus you see we have one veterinarian as a member of the State Board of Health and two others engaged in the field-work of the board. One of these field-veterinarians devotes his entire time to hog-cholera; the other does miscellaneous work, going to outbreaks of any disease of unusual importance, to outbreaks where there is dispute among different veterinarians called by owners and local boards, and to places in the State where there are no competent veterinarians.

Perhaps I should explain that in Minnesota we expect the local board to employ in ordinary cases a local veterinarian, and take care of their own outbreaks of infectious diseases among domestic animals under the direction, of course, of the State board. The law requires that local health officers shall report to the State Board of Health within twenty-four hours after receiving information of an infectious disease.

During the four years of my work as an experiment-station veterinarian, before my connection with the State Board of Health, I was constantly crippled for lack of police authority. An experiment-station veterinarian is usually expected to visit outbreaks, make diagnoses, and write prescriptions, and then he is severely blamed because the outbreak of glanders or anthrax, or, possibly, sheep-scab, does not promptly abate. During this time I could give information and advice and write prescriptions, but had no authority to insist on anything. If I did this kind of work for the State Board of Health the station received no credit.

On the other hand, the State Board of Health veterinarian, or State veterinarian, as the case may be, who has no connection with an experiment station, is very apt to be crippled for lack of opportunities and funds for investigation. For instance, he visits an outbreak of disease that affords a very peculiar and unusual history. The trouble may be due to faulty conditions of the feed, but he is unable to make a careful investigation and gather satisfactory information as to the cause and nature of the trouble, perhaps, for lack of funds for such work.

An experiment station veterinarian, who is also a State Board of Health veterinarian, or State veterinarian, has splendid opportunities for collecting material, for doing a great variety of experimental work, and keeping accurate records, with very little expense to the station. He can collect an abundance of material for almost any sort of experimental work, with little expense to the station. This is especially true if he has access to a well-furnished bacteriological laboratory.

Another advantage is that such an arrangement brings about a hearty coöperation between two great institutions which might otherwise be working separately and more or less fruitlessly in the same field, each one's work incomplete without the data which the other could furnish. By the way, I might suggest that in Minnesota this plan of coöperation in matters of agricultural interest is in quite general and happy operation. For instance, our State University, including our Agricultural College and School of Agriculture, our experiment station, and State farmers' institutes, are all intimately associated in their work, partly because the regents of the University and experiment station are influential members on the board of control of the State farmers' institutes. Our State fair grounds adjoin the experimental farm, and there is the closest possible coöperation between the State Agricultural Society, Minnesota Stockbreeders' Association, and the experiment station, with its congeners, the College and School of Agriculture and the State farmers' institutes.

The experiment-station veterinarian is also director of the veterinary department of the State Board of Health.

We find coöperation between the veterinary work of the experiment station and the State Board of Health to be very satisfactory. We found the work unsatisfactory before such combination was made. So long as we had one authority in the State who had charge of infectious diseases, and another who worked in both parts of this field, but had no police authority for infectious diseases, the work for each outbreak was more or less tangled.

Owing to the way in which the work is organized in Minnesota, outbreaks of infectious diseases among domestic animals are discovered and reported by the local health-officer to the State board. If the outbreak is such that it can be taken care of by the local health-officer or by a representative of the State Baord of Health, and all that is needed is a little police authority, it does not necessarily involve the station-work at all. On the other hand, if it is work that invites investigation, the experiment station furnishes materials and means for such work, and finally, if it is thought best, publishes and distributes.

If representatives of the State Board of Health and experiment station go to the Legislature together and ask for an appropriation or modification of existing laws, they are likely to be successful.

Correspondence and other office-work of the veterinary departments of the two institutions can be greatly economized by coöperation. There is needed only one set of office records and one official head for the two departments. Although there may be a large correspondence and an immense amount of office records and files to look after, the work can be so planned that one office assistant does this work for both. In our State the experiment station permits me to use a portion of my time for the State Board of Health work, on the ground that I would have to do a great deal of this work, whether connected with the State Board of Health or not. The office assistant and stenographer does all my correspondence and keeps station records, although her salary is paid by the State Board of Health.

By this coöperation we avoid a great deal of duplicating which would otherwise be unavoidable; for instance, I write a small bulletin on hog-cholera and swine-plague for the experiment station. After it has been distributed by the experiment station I condense it into a small circular for use in the State Board of Health work.

Let me say, in conclusion, that I hope that the work of this Association may aid in bringing about greater uniformity and closer coöperation between our various States; and when this work is organized as it should be every State will have one or more veterinarians on the State Board of Health, and the station veterinarian will be *ex-officio* a member of that board.

